# Nasal high-flow compared to non-invasive ventilation in treatment of acute acidotic hypercapnic exacerbation of chronic obstructive pulmonary disease—protocol for a randomized controlled noninferiority trial (ELVIS)

**DOI:** 10.1186/s13063-021-05978-z

**Published:** 2022-01-10

**Authors:** Jens Bräunlich, Nicole Köppe-Bauernfeind, David Petroff, Annegret Franke, Hubert Wirtz

**Affiliations:** 1 University of Leipzig, Leipzig, Germany; 2 Hospital Emden, Bolardusstrasse 20, 26721 Emden, Germany

**Keywords:** Chronic obstructive pulmonary disease, COPD, Nasal high-flow, Non-invasive ventilation, Acidotic hypercapnic exacerbation, intubation, Randomized controlled trial, RCT

## Abstract

**Background:**

Acute exacerbations of chronic obstructive pulmonary disease (AECOPD) have a major negative impact on health status, rates of hospitalization, readmission, disease progression and mortality. Non-invasive ventilation (NIV) is the standard therapy for hypercapnic acidotic respiratory failure in AECOPD. Despite its beneficial effects, NIV is often poorly tolerated (11–34 % failure rate). An increasing number of studies have documented a beneficial effect of nasal high-flow (NHF) in acute hypercapnia. We designed a prospective, randomized, multi-centre, open label, non-inferiority trial to compare treatment failure in nasal NHF vs NIV in patients with acidotic hypercapnic AECOPD.

**Methods:**

The study will be conducted in about 35 sites in Germany. Patients with hypercapnic AECOPD with respiratory acidosis (pH < 7.35) will be randomized 1:1 to NIV or NHF. The primary outcome is the combined endpoint of intubation, treatment failure or death at 72 h. The switch from one to the other device marks a device failure but acts as a rescue treatment in absence of intubation criteria. A sample size of 720 was calculated to have 80% power for showing that NHF is non-inferior to NIV with a margin of 8 percentage points. Linear regression will be used for the confirmatory analysis.

**Discussion:**

If NHF is shown to be non-inferior to NIV in acidotic hypercapnic AECOPD, it could become an important alternative treatment.

**Trial registration:**

ClinicalTrials.gov, NCT04881409, Registered on May 11, 2021

**Supplementary Information:**

The online version contains supplementary material available at 10.1186/s13063-021-05978-z.

## Background

Chronic obstructive pulmonary disease (COPD) is a common, preventable and treatable disease characterized by persistent respiratory symptoms and airflow limitation due to airway and/or alveolar abnormalities usually caused by significant exposure to noxious particles or gases. COPD is a leading cause of morbidity and mortality worldwide. Prevalence of COPD is much higher in smokers and ex-smokers, in subjects of ≥ 40 years of age, and in men. Approximately, three million deaths occur annually worldwide. The prevalence of COPD is expected to rise in the decades ahead because of continued exposure to COPD risk factors and ageing of the population [1–3].

The Global Burden of Disease Study reports a prevalence of 251 million cases of COPD in 2016 [1, 4]. 3.17 million deaths were reported to be caused by COPD in 2015 (5% of all deaths). Most patients with COPD develop typical exacerbations of the disease during their lifetime. In severe AECOPD hospitalization is required. Data from the European COPD audit in 13 countries and 422 hospitals [4] monitored 16,016 patients. Of those patients who had blood gas checks at the time of hospital admission (*n* = 13,069), 5933 had hypercapnia (45.4%) and 2452 (18.8%) had respiratory acidosis.

Acute exacerbations of chronic obstructive pulmonary disease (AECOPD) episodes have a major negative impact on health status, rates of hospitalization, readmission, disease progression and mortality [5]. AECOPD is characterized by increased dyspnoea, increased sputum purulence and volume. Severe AECOPD can lead to acute hypercapnic acidotic respiratory failure, a life-threatening condition. The mortality of AECOPD is severe with 183 deaths of 1000 patients with hypercapnic AECOPD and 341 of 1000 will be intubated and mechanically ventilated [5].

Non-invasive ventilation (NIV) is the standard therapy for hypercapnic acidotic respiratory failure in AECOPD [6]. Solid evidence of its effectiveness has been generated for more than two decades with RCTs demonstrating rapid improvement in blood gases, respiratory rate, need for intubation, length of hospital stay and mortality [5–8]. Despite its beneficial effects, NIV is often poorly tolerated (11–34 % failure rate) [5, 9, 10]. In most cases, the adaptation is difficult and time-consuming and may require patient sedation.

Nasal high-flow (NHF) provides warmed and humidified gas administered through larger bore soft nasal prongs. The almost saturated and warmed gas flow is the basis of good tolerance even at high flow rates (30–60 l/min). NHF results in only a small increase in airway pressure (further reduced by opening the mouth). NHF reduces minute volume, lowers respiratory rate and decreases the work of breathing. Exhaled gas in the upper airways is rapidly washed out and thus physiological dead-space is reduced [11–15]. The high flow rates delivered by NHF are sufficient to cover even high peak inspiratory flows, thereby avoiding the admixture of ambient air.

In a recent study, NHF was found to be superior to standard nasal prongs (SNP) and NIV in patients with severe hypoxemic respiratory failure with regard to intubation rates and mortality [16]. The reintubation rate in the NHF arm was non-inferior or better compared to either venturi mask, SNP or NIV respectively [17, 18] in a mixed hypoxemic population. Most data relating to effectiveness of NHF consider post-extubation respiratory failure also including respiratory acidosis defined as pH < 7.35 and PaCO_2_ > 45 mmHg. Interestingly, in the two studies by Hernandez et al., a trend of decreasing respiratory acidosis was reported during NHF therapy [18]. These trends were found in comparison to conventional oxygen and non-invasive ventilation (NIV).

A number of studies and case reports with hypercapnic patients are being reported [11, 12, 14, 19]. Most of these studies investigating the effects of NHF on chronic hypercapnia have been conducted in COPD patients. The first study was the investigation by Bräunlich et al. in 2013 [12]. This mechanistic study was the first to describe the changes in respiratory patterns in healthy volunteers, patients with COPD and lung fibrosis. Patients with stable values of capillary pCO_2_ using NHF for 8 h during the daytime with a flow of 24 l/min showed a decrease in capillary pCO_2_ by 0.69 ± 0.2 kPa [12]. The reduction in hypercapnia was seen despite a decrease in respiratory rate and minute volume. Significant changes were also found in patients with interstitial lung disease (ILD). Another study by our group confirmed these results and documented a decrease in capillary pCO_2_ in 54 COPD patients [20]. A major finding was a greater extent of decarboxylation by using higher flow rates. The mean value decreased from 91 ± 6.7% at 20 l/min to 87.4 ± 6.2% at 30 l/min after a 2-h treatment period. In agreement with the study by Frizzola et al., we observed a decrease in hypercapnia that was a flow-dependent [21]. A study by Pisani et al. investigated patients with COPD and reported a decrease in arterial pCO_2_ at a flow rate of 20 and 30 l/min with the mouth closed. With 30 l/min but not with 20 l/min, a concomitant decrease in pO_2_ was observed with an accompanying decrease in respiratory rate [22]. The retrospective clinical study by Jeong et al. revealed the potential decrease in hypercapnia during NHF therapy in a cohort of 46 patients with and without COPD in an emergency department [23]. Most of the patients in the hypercapnic group had a AECOPD. Hypercapnia decreased significantly with an increase in paO_2_. But this observation was only found in hypercapnic patients.

Our TIBICO trial is a 6 week, cross over study using either NHF or NIV in stable hypercapnic COPD patients without recent exacerbation. The main result was that NHF was similar to NIV in terms of decreasing hypercapnia and quality of life. This was the first randomized controlled trial indicating the stable effect of respiratory support in hypercapnic patients by NHF [14]. Taking this study into account together with CO_2_ wash-out studies, we concluded that AECOPD patients might benefit from NHF as much as from NIV.

Few studies regarding the use of NHF have been published in acute AECOPD. A couple of recent trials appear to confirm our hypothesis in mixed populations with a subset of hypercapnic AECOPD patients with significant improvements in blood gases during NHF therapy [24, 25]. In a pilot study retrospectively looking at acidotic AECOPD patients that did not tolerate prior NIV and prospective RCTs, we and others observed improvements with NHF that were comparable to those generally observed with NIV [19, 26]. The study by Cortegiani et al. investigated patients with acidotic hypercapnic AECOPD. The primary endpoint was the decline in hypercapnia compared to NIV. They found no difference after 2 h between the two devices after randomization of 80 patients [27].

The main objective of the ELVIS clinical trial will be a direct comparison of nasal high flow and non-invasive ventilation (via oral/nasal mask) in hypercapnic, acidotic AECOPD patients.

## Methods

### Study design

ELVIS is an investigator initiated trial funded by the German Ministry for Education and Research (BMBF KS2018-073). In a legal sense, this trial is regulated by the European Medical Devices Law for use of approved devices in their indication. The legal basis for the study is the German Medical Devices Act (MPG §23b), valid before the MPDG came into force. As such, there is cannot be a legal sponsor. However, Leipzig University is the “Responsible Institution,” and neither the university qua university nor the BMBF played any part in study design. Furthermore, they will play no part in data collection, management, analysis and interpretation of data; writing of the report; and the decision to submit the report for publication. The trial is designed as a prospective, randomized, multi-centre, open label, non-inferiority trial. Blinding is not possible because of the different nature of the two different devices, but the endpoints were chosen to be as objective as possible to avoid potential bias. The analysts will not be blinded. The study will be conducted in about 35 university and community hospitals in Germany involving the pneumology and emergency wards as well as ICU; see supplement for the list of intended sites.

Each trial site receives a study-specific NHF device (through public funds), although other specified devices may also be used. The handling of the devices is carried out according to the manufacturer's instructions. The protocol was approved by the Ethics Committee of all participating centres. The study was registered at ClinicalTrials.gov in May 2021 (NCT04881409). Study monitoring, data handling and organization of the study will be provided by Clinical Trials Centre from the University of Leipzig, Germany.

### Study population

Patients are eligible for the study if they suffer from AECOPD in the absence of other causes of respiratory failure. Investigators from the pulmonology or emergency wards or from the intensive care unit will briefly describe the trial and obtain informed consent. A concise consent for is available if treatment has to begin imminently, and the full consent is then required at a later point in time. On the consent form, participants are informed in detail about data handling and storage. This trial does not involve collecting biological specimens for storage.

Specific eligibility criteria are as follows.

*Inclusion criteria*

1. Acute hypercapnic exacerbation of chronic obstructive pulmonary disease with respiratory acidosis (pH < 7.35)
2. pCO_2_ > 45 mmHg
3. age ≥ 18 years
4. Written informed consent

*Exclusion criteria*

1. Immediate need for intubation
2. pH < 7.15
3. BMI ≥ 35 kg/m^2^
4. Established home-NIV or home-CPAP
5. End-stage disease with DNI/DNR order
6. Diseases that could influence the primary endpoint: e.g. acute heart infarction, cardiogenic lung oedema, acute and massive lung embolism (hypertensive), chronic dialysis with metabolic acidosis, unstable rib fracture influencing ventilation, injury to the face prohibiting use of a face mask
7. Acute disease that precludes participation in the trial
8. Tracheotomized patients
9. Psychological/mental or other inabilities to supply required informed consent
10. Participation in other interventional trials
11. Suspected lack of compliance

### Interventions

**Fig. 1:**
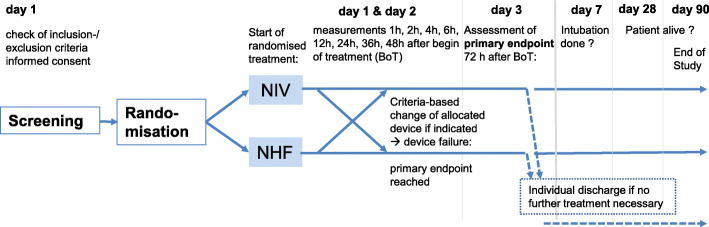
Study flow chart according to the Consolidated Standards of Reporting Trials

Patients will be randomized to two groups (Fig. 1) and will start using the allocated respiratory support device immediately. For nasal high-flow, “TNI soft flow 50 or 60” is recommended and will be provided by the trial centre before the start of the study. However, other dedicated nasal high-flow devices may also be used. Such devices must be capable of a flow of at least 50 l/min as well as sufficient heating up to the prongs and humidification. So-called hybrid devices are not permitted. NHF will start ideally with a flow of 40 l/min at 37° and FiO_2_ of 0.4, generally with standard or large sized prongs. Later adjustments according to oxygen saturation or patient comfort (temperature and humidity) at a patient level are possible.

For patients randomized to the NIV arm, NIV is initialized using a regular oral/nasal mask. Pressure support will be initiated with 18/4 mbar for adaptation and then increased to achieve effective respiratory support while trying not to lose tolerance.

With both devices, oxygen should be supplemented to reach an O_2_ saturation range of 88–92%. Patients should use respiratory support as long as possible, both day and night. The study intervention can be initiated at the emergency room or in intermediate or intensive care or a specialized respiratory medicine ward as mentioned above. The use of either device does not require alteration to usual care pathways (including use of any medication), and these will continue for both trial arms.

Statistical monitoring based on data in the database will take place at regular intervals and include the verification that reasons for switching devices are documented in a timely manner and follow the protocol specification. It will also be used to ensure that investigators provide the duration of device use as a measure of patient compliance. Departures at the site level from protocol specifications will lead to corrective measures.

Patients are covered by insurance while in the trial and after the trial; treatment is at the physician’s discretion but may include the continued use of the prescribed device.

### Study endpoints

The primary endpoint is the proportion of patients with treatment failure within 72 h. Treatment failure is defined by (a) switch to another method of non-invasive ventilation or (b) intubation or (c) death.

It is highly recommended that intubation only be initiated if pH < 7.15 and at least one of the following criteria is met: (i) Glasgow Coma Scale (GCS) < 10, (ii) O_2_ saturation < 85% or PaCO_2_ < 45 mmHg despite FiO_2_ > 0.5 and (iii) respiratory rate > 40 cycles/min.

Switching to another method of non-invasive ventilation should only be considered in the following situation:

- One of the following criteria is met within 1 h: decrease in pH by at least 0.06, increase in pCO_2_ by at least 10 mmHg, unacceptable decrease in GCS, increase in respiratory rate by at least 20%, insufficient compliance
- One of the following criteria is met after 2 h: unacceptable decrease in pH or pCO_2_, clinically unstable compared to last status (beginning at 4 h), insufficient compliance

The most important secondary endpoints are as follows: (a) proportion of patients that fulfil single components of the primary endpoint (i.e. intubation, switch of device or death within 72 h), (b) proportion of patients intubated within 7 days after hospitalization/randomization, (c) overall survival at 28 and 90 days, (d) (invasive) ventilator-free days up to day 28, (e) (invasive) ventilator-free hours until 72 h or until reaching the primary endpoint (whichever comes first), (f) ICU and hospital lengths of stay, (g) proportion of patients requiring sedation and (h) quality of life at day 28 according to St George’s Respiratory Questionnaire (SGRQ) and the Severe Respiratory Insufficiency (SRI) questionnaire, both of which are validated and standard instruments used in patients with obstructive airway diseases [27–29]. Safety issues of interest are device related intolerance/complications and severe diseases acquired under treatment. Compliance and acceptance of devices will also be investigated.

### Randomization, recruitment and data collection

Randomization will be performed centrally, via a secure web-based tool. The allocation to the intervention arm (randomization ratio 1:1) uses a minimization procedure with a stochastic component that takes into account trial centre, pH ≤/> 7.3 and BMI ≤/> 30 kg/m^2^. The randomization result forwarded automatically via email to the investigator and the Clinical Trial Centre Leipzig.

Adequate recruitment will be encouraged through regular Newsletters including current recruitment status, regular consultations with trial sites by the monitor/coordinating investigator and trial meetings.

The schedule of study visits and list of parameters collected can be found in Table 1. Within the first 24 h, data are collected at 1, 2, 4, 6, 12 and 24 h after which collection takes place at 36, 48 and 72 h. There is a discharge visit and one at day 7 as well as telephone visits on days 28 and 90.

**Table 1:** Schedule for study interventions according to Recommendations for Interventional Trials (SPIRIT) indications

Examination/assessment		Treatment											Follow-up		
	Screening	Visit 0	Visit 1						Visit 2		Visit 3		Visit 4	Visit 5^**a**^	Visit 6^**a**^
Timepoint		0 h	1 h ± 30 min	2 h ± 30 min	4 h ± 30 min	6 h ± 30 min	12 h ± 60 min	24 h ± 60 min	36 h ± 90 min	48 h ± 120 min	72 h ± 180 min	discharge	day 7^**b**^ ± 1 day	day 28	day 90
Informed consent	X^c^											X^d^			
Inclusion criteria/exclusion criteria	X														
Randomization		X													
Patient characteristics (anamnesis, comorbidity)	X														
Medication	X							X^e^		X^e^	X^e^	X			
Clinical parameters (heart rate, respiratory rate, blood pressure, Borg’s scale)	X		X	X	X	X	X	X	X	X	X	X	X		
Adverse events/side-effects			X	X	X	X	X	X	X	X	X	X	X	X	X
Glasgow coma scale (GCS)	X		X	X	X	X	X	X	X	X	X	X			
Blood gas analysis (BGA): pO_2_, pCO_2_, pH, SpO_2_, FIO_2_	X		X	X	X	X	X	X	X	X	X	X	X		
Therapy according to allocated device: device parameters		X	X^6^	X	X	X	X	X	X	X	X	X	X^g^	X^f^	X^f^
Therapy: oxygen supplement		X	X	X	X	X	X	X	X	X	X	X	X^f^	X^f^	X^f^
infection parameters and biomarkers (optional)	X							X^e^			X^e^				

^a^Visits 5 and 6 are conducted by telephone

^b^Assessment of intubation; not applicable, if patient is already discharged since no intubation can be stated in this care

^c^Standard (long) version of informed consent OR concise (short) version of informed consent

^d^Standard (long) version of informed consent, if patient is still able to give informed consent

^e^All medications within the last 24 h resp. laboratory parameters any time within the last 24 h

^f^A change of device is possible, if switch criteria are fulfilled OR need for intubation criteria are met before 72 h → primary endpoint reached

^g^Ongoing treatment, if patient still hospitalized

The case report form (CRF) will be designed by the ZKS Leipzig in cooperation with the co-ordinating investigator and provided in electronic form (eCRF). In order to facilitate the documentation as per protocol in case of malfunction of the electronic system or any of its components, a paper version of the CRF (interim CRF) will be provided in the ISF (investigator site file). The data on this paper version will be transferred to the eCRF as soon as the electronic system is available again.

The eCRF must be completed shortly after each trial visit according to ISO 14155:2020 chapter 7.8.1 and to enable central monitoring of the trial data.

Access to the data base will be limited to authorised staff and patients will be identified by patient-ID only. Authorization is granted by the site’s investigator using the trial specific staff signature and delegation log. Based on the staff signature and delegation, log access to the eCRF will be granted by the responsible staff at the ZKS Leipzig. Authorised staff members on site will be able to enter and update data as well as finalise data by electronic signature during the conduct of the trial according to a trial specific concept for documentation. All entries and data changes will be tracked automatically including date, time and person who entered/changed information (audit trail). Major correction(s) or major missing data have to be explained.

Each trial centre is initiated by the clinical monitor before start of the study, which means that the implementation of the study is explained and all necessary documents are provided. During trial conduct, central and statistical monitoring procedures will be combined with on-site monitoring visits in order to achieve high protocol compliance and data quality, as well as to ensure patients’ safety and rights. All clinical data are entered by the investigators (or their designated staff) into electronic data collection forms, which are all available in the database. Access to the data base will be limited to authorised staff only after training on the database.

In case of premature termination of therapy, it is necessary to document the date (as exactly as possible), the reason of termination and the current condition of the patient. Therefore, the eCRF “End of study (ES)” has to be completed for each patient. Data entry to this eCRF page will trigger an automatic report to the responsible trial team members at the ZKS Leipzig.

After a patient has been enrolled, it is the investigator's responsibility to avoid protocol violations in order to obtain unbiased data for the trial. Those protocol violations deemed to be major are defined by the risk analysis performed before and during trial implementation and will be further detailed in separate documents belonging to the risk assessment/monitoring plan. This list can be augmented in the course of the trial. Major protocol violations will be reported to the coordination centre Leipzig, which will inform the co-ordinating investigator. All protocol violations will be documented and discussed with the responsible biometrician before closing the data base and carrying out the statistical analysis. The investigator must ensure that the recorded data are documented as per protocol. Minor variations are inevitable in clinical routine but must be documented together with a justification.

### Protocol modifications

Substantial modifications to the protocol require a formal amendment that has to be approved by the lead Ethics Committee. Furthermore, the changes must be re-appraised by the co-ordinating investigators and, if applicable, in agreement with the biometrician and/or DSMB. Protocol amendments will be communicated by newsletters and at trial meetings but also with each trial site individually by Data Management after the local Ethics Committee has approved it. The Investigator Site File will be updated and all relevant changes to recruitment, clinical procedures and data entry will be discussed.

### Sample size and statistical analysis

We followed statistical guidelines, recommending that clinical and statistical considerations be taken into account along with historical data when choosing a margin of non-inferiority [30]. Moreover, we note that the European Medicals Agency (EMA) and the Food and Drug Administration (FDA) often find proposed margins too lax and chose a stricter margin than the 15 and 10 percentage points used in two RCTs with non-inferiority designs for ventilation failure [5, 18, 24]. The point estimate from historical data for absolute risk reduction in intubation proportions due to NIV was found to be 20.7 and 11.9 with lower 95% confidence limits of 2.0 and 1.6 percentage points, respectively [5, 7, 8]. Taken all this into consideration, we chose Δ = 8 percentage points for our non-inferiority margin. The cited trials by Plant et al. and Carrera et al. found need for intubation in 13.5% and 15.1% of the NIV groups. We use this as a proxy for our endpoint and assuming that treatment failure is 15% in both arms, implying that 680 patients need to be analysed to have 80% power for having the upper end of the 95% CI within the margin of non-inferiority (continuity corrected *Z*-test with pooled variance) [31]. Taking into account a small drop-out rate of about 5%, we intend to recruit 720 patients in total.

The full analysis set (FAS, based on the intention-to-treat (ITT) strategy) is defined to comprise all randomized patients with AECOPD and started on ventilatory support. If, for example, pneumonia is detected within 48 h (nosocomial), such patients will not be included in the final analysis, since the initial AECOPD diagnosis is found to be erroneous. Patients who test positive for SARS-CoV-2 within 48 h will not be included in the full analysis set. For the confirmatory analysis, linear regression with the stratification variables and the arm as covariates will be used and where the dependent variable will be coded as 0, 1. A 95% two-sided Wald confidence interval for the arm term can then be interpreted in terms of absolute risk and the null hypothesis is rejected if it does not cross the non-inferiority margin. As a sensitivity analysis, a two-sided 95%-Wilson confidence interval for the difference in proportions will be calculated. Only few missing data regarding all relevant endpoints are expected given the rather short period of observation. Nevertheless, conservative imputations will be performed independent of treatment arm. Further sensitivity analyses are planned to adjust for covariates, possibly imbalanced baseline characteristics between groups and/or protocol deviations in multivariable regression models, e.g. for (components of) the primary endpoint and/or in subgroups, which arose from stratification criteria. Furthermore, the odds ratios from logistic regression will be computed to have a relative risk measure in addition to the absolute one, as recommended by statistical guidelines.

Absolute risk differences of major secondary endpoints will be analysed in the same way as the primary endpoint. Kaplan-Meier curves/results of logrank tests will be presented for 28 and 90 days mortality. We expect the time to death to be (nearly) always available. Ventilator-free hours (until 72 h assessment) and SRI and SGRQ at 28 days will be analysed by a linear regression model including randomization arm as factor and stratification criteria as covariates. SRI and SGRQ cannot be determined at baseline due to the patients’ state.

Neither imputation of missing values nor adjustments for multiple testing are planned for secondary/safety endpoints. No interim analysis is planned.

The proportion of patients (with 95% confidence limits) who were randomly allocated to NIV therapy but changed to NHF due to insufficient efficacy and avoid an immediate intubation and vice versa will be analysed. Both the proportion with and without later intubation will be provided. Although these NIV-to-NHF proportions are a form of data exploration, useful estimates on the value of NHF as rescue treatment in patients who do not tolerate NIV may be derived, especially if compared to usually reported proportions of intubation immediately initiated after NIV.

The proportion of switches to another device before intubation will also be compared to investigate potentially existing preferences in favour of NIV.

### Roles and responsibilities

The Leipzig University as the responsible institution, together with ZKS Leipzig (coordinating centre) and the trial sites, is responsible for the implementation and data processing in accordance with Article 4(7) of the General Data Protection Regulation 2016/679 in this trial. The ZKS Leipzig is responsible for implementation of procedures for data collection, storage, protection, retention and destruction. The data stored in the trial database is secured against unauthorized access.

The study is supervised by a project manager in close cooperation with the coordinating investigators, who takes care of all regulatory and organizational processes and monitors the conduct of the study. In addition, the study team consists of data management, clinical monitoring and biometric staff. The data management team monitors the electronically entered data and makes inquiries to the clinics if necessary. Clinical monitors appointed by ZKS Leipzig visit the recruiting sites regularly and verify the informed consent forms. This serves to verify that the patient has unambiguously given his or her consent for trial participation as well as for data capture, transmission and analysis. The patients are informed of this fact and agree to the procedure with the patient information/informed consent.

There is no trial steering committee.

### Harms

All adverse events will be documented on special AE-forms from start of ventilation until discharge or day 28 (whichever comes first) for each patient. Information relevant to AEs will be solicited by the investigator at every patient’s study visit. In addition, the patient will be asked to inform the clinical trial site of any health problems arising between visits by phone or personal visit. Adverse events are classified by their seriousness, intensity and relationship to the therapeutic intervention.

If an AE fulfils any of the criteria for a SAE, the AE has to be marked as “serious” on the CRF. This applies to all SAEs, whether or not they are considered to be related to the study treatment.

For both serious and non-serious AEs, documentation should be supported by an entry in the patient’s health record. Required information includes the type of AE, seriousness of the event, an estimate of its severity, start date, date of resolution, actions required, outcome and an assessment of its relationship to trial intervention. All abnormal physical and/or laboratory results which are considered to be clinically relevant by the investigator should be recorded as (S)AEs. The investigator will follow-up the event until the AE has been resolved, resolved with sequelae, or was fatal. The investigator should report each AE on according eCRF in a timely manner and continuously during the trial. Within the database, a selection of the radio button “serious” will trigger an automatic e-mail-announcement of the SAE at ZKS Leipzig. If any of the involved ethics committees should require SAE-reports, these will be derived from the trial database at the required intervals. The ELVIS trial follows §23b MPG. Thus, there are no SAE-reporting obligations to the competent authority.

### Trial oversight

The ZKS Leipzig will be responsible for trial monitoring. Initiation, regular and close-out visits will be performed in all trial sites. A risk-based monitoring strategy will be implemented, as required by ISO 14155: 2020. During trial conduct, central and statistical monitoring procedures will be combined with on-site monitoring visits in order to achieve high protocol compliance and data quality, as well as to ensure patients’ safety and rights. The chosen monitoring strategy depends on the results of the risk analysis done during the protocol development and will described in the trial specific monitoring plan.

In general, a first monitoring visit at a trial site will be scheduled after the inclusion of the site’s first three patients, checking protocol compliance and preventing further systematic errors due to misunderstandings. All trial sites will then be visited regularly. The frequency of further on-site monitoring visits will depend on the trial site’s recruitment rate and on whether problems have been detected with the site, either by prior on-site visits or by central monitoring.

The responsible institution might conduct site audits in order to guarantee that the conduct of the trial is in accordance with the DoH, DIN ISO 14155 and the trial protocol. The investigator agrees to provide access to the auditor for all relevant documents.

An Independent Data Safety and Monitoring Board (DSMB) consisting of two clinicians and an expert in medical statistics will meet periodically to perform a review and an evaluation of the accumulated study data. The DSMB is responsible for reviewing safety of the trial intervention, integrity and validity of the data, appropriate study conduct and study progress.

### Dissemination plan and authorship

Trial progress will be communicated internally via newsletters and consortium meetings. The trial results will be disseminated at national and international conferences and published in English language journals. Authorship will follow the criteria developed by the International Committee of Medical Journal Editors (ICMJE), including those that distinguish authors from other contributors. There is no plan to use professional writers. Upon publication of the main results, we plan to make the full trial protocol and statistical analysis plan publically available, most likely as supplementary material. Individual patient data may be shared after de-identification if the researcher requesting data has local ethics committee approval and has publically registered the planned analysis. Informed consent forms and case report forms may be made available upon reasonable request.

## Discussion

Guidelines recommend the use of NIV as standard therapy in acidotic hypercapnic AECOPD. NIV has been shown to prevent intubation and reduce mortality [6]. However, in broad clinical practice its use is neither widespread nor routine [4]. The reasons range from lack of expertise, unavailable devices or shortage of staff. Up to 30% of AECOPD patients do not tolerate NIV in acute setting.

NHF has been shown to be effective in different settings and is strongly recommended in acute hypoxemic respiratory failure [32, 33]. Following extubation and in the subsequent time period NHF has been recommended [6]. There is growing interest in the use of NHF in ventilatory failure, but most RCTs in this setting have excluded hypercapnic patients [16–18]. It is therefore not surprising that in these studies the investigators found only small decreases in pCO_2_. Today, there is growing evidence that NHF results in pCO_2_ reduction in hypercapnic patients over shorter time periods [11, 12, 20]. In a small pilot trial in patients with stable hypercapnic COPD over 6 weeks, NHF was found to be not inferior compared to NIV in reducing pCO_2_ [34]. The first randomized controlled trial in chronic hypercapnic respiratory insufficient COPD patients strongly suggested non-inferiority of NHF compared to NIV in terms of decarboxylation and some scales measuring quality of life also showed improvement with NHF [14].

In acute hypercapnic respiratory failure, first data exist, but either study populations were small, inhomogeneous, and most were retrospective without randomized control groups. Comparison to other ventilatory support devices in those areas where NIV is meanwhile well established and recommended by guidelines is lacking. The first randomized controlled trial regarding this aspect was the study by Cortegiani et al. [35] comparing NIV and NHF in 80 hypercapnic patients. The result of this study was non-inferiority of NHF in terms of pCO_2_ reduction, though a non-inferiority margin of 10 mmHg may be considered large.

Because of these encouraging results, a study comparing NIV and NHF AECOPD, a major stronghold of NIV, is timely and necessary. We conceived the study in 2017 and submitted the original draft to the German Ministry for Education and Research in February 2018 in a two-stage process. Since then, a further group has also recognized the need for such a trial and designed a similar one [PMID: 33123554]. There are some differences between their trial and ours regarding specifics of patient population and the definition of treatment failure, but also in the margin of non-inferiority (they choose 10% compared to our 8%), meaning that our sample size is about 220 patients larger and that the interpretation of the results in more stringent in our case. It will be of great interest to compare the results of these two trials and the possibility of combining them in a meta-analysis suggests itself.

Our trial has its limitations. First, blinding is not be possible because of the different nature of the two respiratory support methods. Second, criteria to switch from one to another device or to intubate have been prescribed as clearly as possible, but an element of individual clinical decision is unavoidable und is not always apparent in the CRF documentation. Third, participating centres have expertise in using NIV, but less so with NHF, which could introduce a learning bias.

The results of our study are expected to improve treatment of acidotic hypercapnic AECOPD patients and will provide valuable data especially considering the large sample size.

Trial status: protocol date 16 December 2020. Version final 1.0. The study is currently recruiting. The first patient was enrolled in May 2021. Completion is expected for February 2024.

## Supplementary Information

**Additional file 1.** SPIRIT 2013 Checklist: Recommended items to address in a clinical trial protocol and related documents

**Additional file 2.** ELVIS participating sites

## Data Availability

Any data required to support the protocol can be supplied on reasonable request.

## Abbreviations

(AE)COPD: (acute exacerbation) of chronic obstructive pulmonary disease
NIV: Non-invasive ventilation
NHF: Nasal high-flow
SNP: Standard nasal prongs
ILD: Interstitial lung disease
RCT: Randomized controlled trial
CPAP: Continuous positive airway pressure
DNI/DNR: Do not intubate/do not resuscitate
FiO2: Fraction of inspired oxygen
GCS: Glasgow Coma Scale
SGRQ: St. Georg Respiratory Questionnaire
SRI: Severe Respiratory Insufficiency Questionnaire
ICU: Intensive care unit
BMI: Body mass index
