# Supplementary material for: Nasal high-flow compared to non-invasive ventilation in treatment of acute acidotic hypercapnic exacerbation of chronic obstructive pulmonary disease—protocol for a randomized controlled noninferiority trial (ELVIS)
Source: Trials. 2022 Jan 10;23:28. doi: 10.1186/s13063-021-05978-z (PMC8744018; doi:10.1186/s13063-021-05978-z)
Supplement: Supplementary file 1 — Additional file 1. SPIRIT 2013 Checklist: Recommended items to address in a clinical trial protocol and related documents [file 13063_2021_5978_MOESM1_ESM.docx]

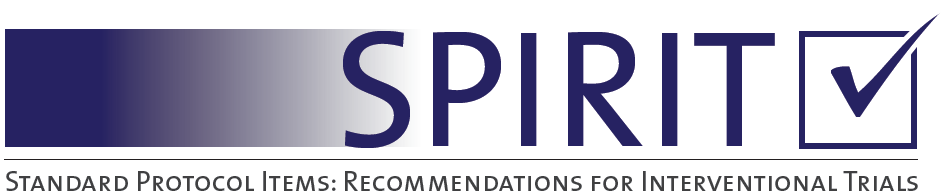


SPIRIT 2013 Checklist: Recommended items to address in a clinical trial protocol and related documents*

| Section/item | ItemNo | Description |
| --- | --- | --- |
| **Administrative information** | | |
| Title | 1 | Descriptive title identifying the study design, population, interventions, and, if applicable, trial acronym  **Provided on page 1** |
| Trial registration | 2a | Trial identifier and registry name. If not yet registered, name of intended registry  **Provided on page 3: ClinicalTrials.gov, NCT04881409** |
|  | 2b | All items from the World Health Organization Trial Registration Data Set  **Please refer to Item 2a and registration in ClinicalTrials.gov, NCT04881409** [**https://clinicaltrials.gov/ct2/show/NCT04881409**](https://clinicaltrials.gov/ct2/show/NCT04881409) |
| Protocol version | 3 | Date and version identifier  **Provided on page 13** |
| Funding | 4 | Sources and types of financial, material, and other support  **Provided on page 2: German Ministry for Education and Research (BMBF KS2018-073)** |
| Roles and responsibilities | 5a | Names, affiliations, and roles of protocol contributors  **Provided on pages 1 and 2** |
|  | 5b | Name and contact information for the trial sponsor  **In a legal sense, this trial is regulated by the European Medical Devices Law for use of approved devices in their indication. As such, there is cannot be a legal sponsor. However, Leipzig University is the “Responsible Institution“.** |
|  | 5c | Role of study sponsor and funders, if any, in study design; collection, management, analysis, and interpretation of data; writing of the report; and the decision to submit the report for publication, including whether they will have ultimate authority over any of these activities  **Neither the university qua university nor the BMBF played any part in study design. Furthermore, they will play no part in data collection, management, analysis, and interpretation of data; writing of the report; and the decision to submit the report for publication . The trial is a publicly funded IIT (manuscript page 5).** |
|  | 5d | Composition, roles, and responsibilities of the coordinating centre, steering committee, endpoint adjudication committee, data management team, and other individuals or groups overseeing the trial, if applicable (see Item 21a for data monitoring committee)  **Provided under Roles and Responsibilities on pages 10-11.** |
| Introduction |  |  |
| Background and rationale | 6a | Description of research question and justification for undertaking the trial, including summary of relevant studies (published and unpublished) examining benefits and harms for each intervention  **Provided on pages 4 and 5** |
|  | 6b | Explanation for choice of comparators  **Provided on pages 4 and 5** |
| Objectives | 7 | Specific objectives or hypotheses  **Provided on page 7** |
| Trial design | 8 | Description of trial design including type of trial (eg, parallel group, crossover, factorial, single group), allocation ratio, and framework (eg, superiority, equivalence, noninferiority, exploratory)  **Provided on pages 5 and 6** |
| Methods: Participants, interventions, and outcomes | | |
| Study setting | 9 | Description of study settings (eg, community clinic, academic hospital) and list of countries where data will be collected. Reference to where list of study sites can be obtained  **Provided on page 6 and in the supplement.** |
| Eligibility criteria | 10 | Inclusion and exclusion criteria for participants. If applicable, eligibility criteria for study centres and individuals who will perform the interventions (eg, surgeons, psychotherapists)  **Provided on page 6** |
| Interventions | 11a | Interventions for each group with sufficient detail to allow replication, including how and when they will be administered  **Provided on page 7** |
|  | 11b | Criteria for discontinuing or modifying allocated interventions for a given trial participant (eg, drug dose change in response to harms, participant request, or improving/worsening disease)  **Provided on page 7** |
|  | 11c | Strategies to improve adherence to intervention protocols, and any procedures for monitoring adherence (eg, drug tablet return, laboratory tests)  **Statistical monitoring is used to detect departures from protocol specifications (details on page 7).** |
|  | 11d | Relevant concomitant care and interventions that are permitted or prohibited during the trial  **We specify that deviations from standard treatments are not needed (page 7).** |
| Outcomes | 12 | Primary, secondary, and other outcomes, including the specific measurement variable (eg, systolic blood pressure), analysis metric (eg, change from baseline, final value, time to event), method of aggregation (eg, median, proportion), and time point for each outcome. Explanation of the clinical relevance of chosen efficacy and harm outcomes is strongly recommended  **Provided on pages 7 and 8** |
| Participant timeline | 13 | Time schedule of enrolment, interventions (including any run-ins and washouts), assessments, and visits for participants. A schematic diagram is highly recommended (see Figure)  **Provided in Table 1** |
| Sample size | 14 | Estimated number of participants needed to achieve study objectives and how it was determined, including clinical and statistical assumptions supporting any sample size calculations  **Provided on pages 9 and 10** |
| Recruitment | 15 | Strategies for achieving adequate participant enrolment to reach target sample size  **Provided on page 8** |
| **Methods: Assignment of interventions (for controlled trials)** | | |
| Allocation: |  |  |
| Sequence generation | 16a | Method of generating the allocation sequence (eg, computer-generated random numbers), and list of any factors for stratification. To reduce predictability of a random sequence, details of any planned restriction (eg, blocking) should be provided in a separate document that is unavailable to those who enrol participants or assign interventions  **Provided on page 8** |
| Allocation concealment mechanism | 16b | Mechanism of implementing the allocation sequence (eg, central telephone; sequentially numbered, opaque, sealed envelopes), describing any steps to conceal the sequence until interventions are assigned  **Provided on page 8** |
| Implementation | 16c | Who will generate the allocation sequence, who will enrol participants, and who will assign participants to interventions  **Provided on page 8** |
| Blinding (masking) | 17a | Who will be blinded after assignment to interventions (eg, trial participants, care providers, outcome assessors, data analysts), and how  **On pages 6 and 13 there is a discussion explaining that blinding is not possible and constitutes a limitation and we specify on page 6 that the analysts will not be blinded.** |
|  | 17b | If blinded, circumstances under which unblinding is permissible, and procedure for revealing a participant’s allocated intervention during the trial  **The design is open label with objective outcomes abstracted from electronic data so unblinding will not occur.** |
| **Methods: Data collection, management, and analysis** | | |
| Data collection methods | 18a | Plans for assessment and collection of outcome, baseline, and other trial data, including any related processes to promote data quality (eg, duplicate measurements, training of assessors) and a description of study instruments (eg, questionnaires, laboratory tests) along with their reliability and validity, if known. Reference to where data collection forms can be found, if not in the protocol  **Provided on pages 8 and 9** |
|  | 18b | Plans to promote participant retention and complete follow-up, including list of any outcome data to be collected for participants who discontinue or deviate from intervention protocols  **Provided on pages 8 and 9** |
| Data management | 19 | Plans for data entry, coding, security, and storage, including any related processes to promote data quality (eg, double data entry; range checks for data values). Reference to where details of data management procedures can be found, if not in the protocol  **Provided on pages 8 and 9** |
| Statistical methods | 20a | Statistical methods for analysing primary and secondary outcomes. Reference to where other details of the statistical analysis plan can be found, if not in the protocol  **Provided on pages 9 and 10** |
|  | 20b | Methods for any additional analyses (eg, subgroup and adjusted analyses)  **Provided on pages 9 and 10** |
|  | 20c | Definition of analysis population relating to protocol non-adherence (eg, as randomised analysis), and any statistical methods to handle missing data (eg, multiple imputation)  **Provided on page 10** |
| **Methods: Monitoring** | | |
| Data monitoring | 21a | Composition of data monitoring committee (DMC); summary of its role and reporting structure; statement of whether it is independent from the sponsor and competing interests; and reference to where further details about its charter can be found, if not in the protocol. Alternatively, an explanation of why a DMC is not needed  **Provided on page 12** |
|  | 21b | Description of any interim analyses and stopping guidelines, including who will have access to these interim results and make the final decision to terminate the trial  **Provided on page 10** |
| Harms | 22 | Plans for collecting, assessing, reporting, and managing solicited and spontaneously reported adverse events and other unintended effects of trial interventions or trial conduct  **Provided on page 11** |
| Auditing | 23 | Frequency and procedures for auditing trial conduct, if any, and whether the process will be independent from investigators and the sponsor  **Provided on page 11 and 12** |
| Ethics and dissemination | | |
| Research ethics approval | 24 | Plans for seeking research ethics committee/institutional review board (REC/IRB) approval  **Approval received, page 2** |
| Protocol amendments | 25 | Plans for communicating important protocol modifications (eg, changes to eligibility criteria, outcomes, analyses) to relevant parties (eg, investigators, REC/IRBs, trial participants, trial registries, journals, regulators)  **Provided on page 9** |
| Consent or assent | 26a | Who will obtain informed consent or assent from potential trial participants or authorised surrogates, and how (see Item 32)  **Provided on page 6** |
|  | 26b | Additional consent provisions for collection and use of participant data and biological specimens in ancillary studies, if applicable  **On page 6 we specify that biological specimens are not collected** |
| Confidentiality | 27 | How personal information about potential and enrolled participants will be collected, shared, and maintained in order to protect confidentiality before, during, and after the trial  **Basics provided on pages 6 and 9** |
| Declaration of interests | 28 | Financial and other competing interests for principal investigators for the overall trial and each study site  **Provided on page 2** |
| Access to data | 29 | Statement of who will have access to the final trial dataset, and disclosure of contractual agreements that limit such access for investigators  **Provided on page 12.** |
| Ancillary and post-trial care | 30 | Provisions, if any, for ancillary and post-trial care, and for compensation to those who suffer harm from trial participation  **Provided on page 7.** |
| Dissemination policy | 31a | Plans for investigators and sponsor to communicate trial results to participants, healthcare professionals, the public, and other relevant groups (eg, via publication, reporting in results databases, or other data sharing arrangements), including any publication restrictions  **Provided on page 10.** |
|  | 31b | Authorship eligibility guidelines and any intended use of professional writers  **Provided on page 12 for the results of the trial and page 13 for the author contributions of the current manuscript.** |
|  | 31c | Plans, if any, for granting public access to the full protocol, participant-level dataset, and statistical code  **Provided on page 12** |
| Appendices |  |  |
| Informed consent materials | 32 | Model consent form and other related documentation given to participants and authorised surrogates  **Statement on availability on page 12.** |
| Biological specimens | 33 | Plans for collection, laboratory evaluation, and storage of biological specimens for genetic or molecular analysis in the current trial and for future use in ancillary studies, if applicable  **None collected in this trial.** |

*It is strongly recommended that this checklist be read in conjunction with the SPIRIT 2013 Explanation & Elaboration for important clarification on the items. Amendments to the protocol should be tracked and dated. The SPIRIT checklist is copyrighted by the SPIRIT Group under the Creative Commons “[Attribution-NonCommercial-NoDerivs 3.0 Unported](http://www.creativecommons.org/licenses/by-nc-nd/3.0/)” license.
