# Supplementary material for: Nasal high-flow compared to non-invasive ventilation in treatment of acute acidotic hypercapnic exacerbation of chronic obstructive pulmonary disease—protocol for a randomized controlled noninferiority trial (ELVIS)
Source: Trials. 2022 Jan 10;23:28. doi: 10.1186/s13063-021-05978-z (PMC8744018; doi:10.1186/s13063-021-05978-z)
Supplement: Supplementary file 2 — Additional file 2. ELVIS participating sites [file 13063_2021_5978_MOESM2_ESM.pdf]

ELVIS - list of participating trial sites

sequential number	trial sites (city)	adress of trial sites	contact person
1	Leipzig	Universitätsklinikum Leipzig<br>Medizinische Klinik und Poliklinik I, Abt. Pneumologie<br>Liebigstr. 20, 04103 Leipzig	Prof. Hubert Wirtz
2	Emden	Klinikum Emden - Hans-Susemihl-Krankenhaus<br>gemeinnützige GmbH<br>Bolardusstraße 20, 26721 Emden	Dr. Jens Bräunlich
3	Berlin	Evangelische Lungenklinik<br>Klinik für Pneumologie<br>Lindenberger Weg 27, 13125 Berlin	Prof. Christian Grohé
4	Dresden	Universitätsklinikum Dresden<br>Medizinische Klinik I, Abteilung Pneumologie<br>Fetscherstr. 74, 01307 Dresden	Prof. Dr. med. Dirk Koschel
5	Aurich	Ubbo-Emmius-Klinik gGmbH<br>Ostfriesisches Krankenhaus, Sektion Pneumologie<br>Wallinghausener Strasse 8-12, 26603 Aurich	Dr. Birgit Pensel
6	Gera	SRH Wald-Klinikum Gera GmbH<br>Pneumologie/Infektologie, Hämatologie/Onkologie<br>Straße des Friedens 122, 07548 Gera	Dr. Horst Zwingenberger
7	Göttingen	Evangelisches Krankenhaus Göttingen-Weende gGmbH<br>Pneumologie, Beatmungsmedizin/Schlaflabor<br>An der Lutter 24, 37075 Göttingen	Prof. Torsten Körber
8	Greifswald	Universitätsklinikum Greifswald<br>Klinik und Poliklinik für Innere Medizin B; Pneumologie<br>Ferdinand-Sauerbruch-Straße, 17475 Greifswald	Dr. Alexander Heine
9	Hamburg-Harburg	Asklepios Klinikum Harburg<br>Klinik für Atemwegs-, Lungen- und Thoraxmedizin<br>Intensivmedizin und Beatmungsmedizin<br>Eißendorfer Pferdeweg 52, 21075 Hamburg	Dr. Martin Bachmann

10	Hemer	Lungenklinik Hemer<br>Zentrum für Pneumologie und Thoraxchirurgie<br>Pneumologie - Intensiv- und Beatmungsmedizin & Schlafmedizin<br>Theo-Funccius-Straße 1, 58675 Hemer	Dr. Michael Westhoff
11	Immenhausen	Lungenfachklinik Immenhausen<br>Zentrum für Pneumologie<br>Robert-Koch-Straße 3, 34376 Immenhausen	Prof. Stefan Andreas
12	Kassel	Marienkrankenhaus Kassel gGmbH<br>Innere Medizin, Pneumologie<br>Marburger Straße 85, 34127 Kassel	PD Dr. Andreas Bastian
13	Oldenburg (Holstein)	Sana Kliniken Ostholstein<br>Klinik Oldenburg<br>Abt. Innere Medizin und Pneumologie<br>Mühlenkamp 5, 23758 Oldenburg in Holstein	Dr. Iris Koper
14	Rosenheim	Klinikum Rosenheim<br>Medizinische Klinik III, Pneumologie<br>Pettenkoferstr. 10, 83022 Rosenheim	Prof. Stefan Budweiser
15	Schmallenberg	Fachkrankenhaus Kloster Grafschaft GmbH<br>Pneumologie und Intensivmedizin<br>Annostraße 1, 57392 Schmallenberg	PD Dr. Dominic Dellweg
16	Solingen	Krankenhaus Bethanien<br>Klinik für Pneumologie und Allergologie<br>Aufderhöher Straße 169-175, 42699 Solingen	Prof. Winfried Randerath
17	Stuttgart (Gerlingen)	Robert-Bosch-Krankenhaus<br>Klinik Schillerhöhe<br>Abtl. Für Pneumologie und Beatmungsmedizin<br>Solitudestraße 18, 70839 Gerlingen	Prof. Claus Neurohr
18	Würzburg	Klinikum Würzburg Mitte gGmbH<br>Standort Missioklinik<br>Pneumologie und Beatmungsmedizin<br>Salvatorstr. 7, 97074 Würzburg	Dr. Stefan Baron

19	Aachen	Uniklinik RWTH Aachen<br>Klinik für Pneumologie und Internistische Intensivmedizin<br>(Medizinische Klinik V)<br>Pauwelsstraße 30, 52074 Aachen	Prof. Michael Dreher
20	Gauting	Asklepios Fachklinik München-Gauting<br>Abt. Intensiv-, Schlaf- und Beatmungsmedizin<br>Robert-Koch-Allee 2, 82131 Gauting	Dr. Lorenz Nowak
21	Halle/Westfalen	Klinikum Halle/ Westf.<br>Klinik für Pneumologie, Schlaf- und Beatmungsmedizin<br>Winnebrockstr. 1, 33790 Halle (Westfalen)	Dr. Jörg Schmitthenner
22	Borstel	Medizinische Klinik Borstel<br>Leibniz Lungenzentrum, Praxis Pneumologie<br>Parkallee 35, 23845 Borstel	Dr. Antonia Sassmann-Schweda
23	Bad Berka	Zentralklinik Bad Berka<br>Klinik für Pneumologie<br>Robert-Koch-Allee 9, 99437 Bad Berka	Dr. Michael Weber
24	Großhansdorf	LungenClinic Großhansdorf<br>Fachabteilung Pneumologie<br>Wöhrendamm 80, 22927 Großhansdorf	Dr. B. Schucher
25	Hannover	KRH Klinikum Siloah<br>Klinik für Pneumologie, Intensiv- und Schlafmedizin<br>Stadionbrücke 4, 30459 Hannover	Prof. Thomas Fühner
26	Konstanz	Klinikum Konstanz<br>II. Medizinische Klinik - Pneumologie<br>Mainaustraße 35, 78464 Konstanz	Prof. Hans-Joachim Kabitz
27	Rostock	Universitätsmedizin Rostock<br>Zentrum für Innere Medizin<br>Klinik und Poliklinik für Innere Medizin<br>Abteilung Pneumologie<br>Ernst-Heydemann-Str. 6, 18057 Rostock	Prof. Johann Christian Virchow

28	Berlin	Charité – Universitätsmedizin Berlin<br>Medizinische Klinik mit Schwerpunkt Infektiologie und Pneumologie<br>Augustenburger Platz 1, 13353 Berlin	Prof. Norbert Suttorp
29	Wangen	Waldburg-Zeil Kliniken<br>Fachkliniken Wangen<br>Klinik für Pneumologie<br>Am Vogelherd 14, 88239 Wangen/Allgäu	Dr. Dominik Harzheim
30	Braunschweig	Städtisches Klinikum Braunschweig<br>Pneumologie und Beatmungsmedizin<br>Salzdahlumer Str. 90, 38126 Braunschweig	PD Dr. Thomas Bitter
31	Ostercappeln	Niels-Stensen-Kliniken<br>Krankenhaus St. Raphael<br>Klinik für Pneumologie und Beatmungsmedizin<br>Bremer Straße 31, 49175 Ostercappeln	Dr. med. Christoph Hünermann
32	Bremen	Klinik für Pneumologie und Beatmungsmedizin<br>Klinikum Bremen-Ost<br>Züricher Str. 40, 28325 Bremen	Dr. Henriette Berger
33	München	München-Klinik Bogenhausen<br>Englschalkinger Str. 77 1925 München	Prof. Dr. Christoph Dodt
34	Hamburg	Asklepios Klinik Barmbek<br>Rübenkamp 220, 22307 Hamburg	Dr. Forian Bornitz
35	Donaustauf	Klinik Donaustauf<br>Ludwigstraße 68, 93093 Donaustauf	Prof. Dr. med. Michael Pfeifer
